# Global perspectives on surgical instrument fit: unveiling challenges faced by urologists

**DOI:** 10.1007/s00345-026-06747-w

**Published:** 2026-09-03

**Authors:** Sarah Razavi, Emeka Udedibia, Kristin L. Chrouser, Hamid Norasi, M. Susan Hallbeck, Madeline C. Snipes, Jeet Bhavsar, David Hoenig, Zeph Okeke

**Affiliations:** 1 Department of Urology, Wake Forest University School of Medicine, 1 Medical Center Boulevard, Winston-Salem, NC 27157 USA; 2 Department of Urology, Zucker School of Medicine at Hofstra/Northwell, Northwell - North Shore University Hospital, New Hyde Park, NY USA; 3 Department of Urology, University of Michigan, Ann Arbor, MI USA; 4 Department of Surgery, Urology Section, VA Ann Arbor Healthcare System, Ann Arbor, MI USA; 5 Robert D. and Patricia E. Kern Center for the Science of Healthcare Delivery, Mayo Clinic, Rochester, MN USA; 6 Northwell, New Hyde Park, NY USA

**Keywords:** Surgical ergonomics, Urologist, Gender equity, Glove fit, Instrument design

## Abstract

**Introduction:**

To address the limited exploration of surgical ergonomics and instrument fit in urology, we evaluated the relationship between surgeons’ hand size, glove fit, gender, and their perceived ease of using various urologic instruments.

**Methods:**

An anonymous web-based survey was designed and distributed to members of the Endourology Society, Society of Women in Urology, and urology residency programs in the United States. It included demographics, perceived hand size/strength, ease of instrument use, and discomfort.

**Results:**

Among 277 respondents (55% female, mean age 43.0 ± 11.4), 26% reported glove fit issues—more common in females (32% vs. 16%, *p* < 0.001). Nearly half (49%) reported hand size impacted their ability to use instruments and 52.73% reported having to make intraoperative adjustments in order to use the instruments effectively. Female urologists found all surgical instruments harder to use compared to their male counterparts (*p* < 0.05). Overall, 40% of respondents reported using some form of intervention to address discomfort and/or injury related to surgical instrument use (47% of females vs. 34% of males; *p* < 0.05). Female urologists and urologists with smaller hands reported significantly more discomfort using the flexible ureteroscope, disposable flexible ureteroscope, nephroscope, stapler, Ligasure, laparoscopic right angle, Trilogy™ and ShockPulse™ (*p* < 0.05).

**Conclusions:**

Our findings suggest that currently available urologic instruments do not fit a large subset of urologists. Industry collaboration is needed to address ergonomic disparities.

## Introduction

The landscape of surgery has significantly changed with the advent of new technologies. Urology has remained at the forefront of surgical innovation by developing and adopting new techniques to improve patient outcomes. Today, urologists use a variety of surgical instruments to perform various procedures such as open, endoscopic, laparoscopic and robotic surgeries.

Instrument fit generally refers to how well a surgical instrument’s design align with the surgeon’s comfort allowing for efficient handling of instrument during procedures [1, 2]. It has been studied in different surgical disciplines. In one of the initial studies on surgical instrument fit in the era of minimally invasive surgery, Berguer et al. reported hand size as a significant determinant of difficulty using laparoscopic instruments [3]. Subsequent studies confirmed these findings; up to 70% of surgeons report difficulties using surgical instruments. Specifically, female and smaller-handed surgeons were more likely to report struggling with handheld instruments [4, 5]. Glove fit is another important ergonomic concern that can potentially affect use of surgical instruments; poor-fitting gloves can cause reduced grip strength, numbness, limited hand motions, loss of tactile feedback, pain and musculoskeletal discomfort [6]. Glove fit has rarely been explored among surgeons, particularly urologists.

Ease of instrument use also affects the ability to learn a procedure as well as choice of procedure [2]. Beyond causing musculoskeletal injury and creating barriers to surgical education, poor surgical ergonomics contributes to long term health consequences, compromised patient care, early retirement and burnout, particularly among female urologists [7, 8].

Surgical ergonomics and instrument fit in particular, have rarely been systematically explored in urology, especially among female urologists. As the urology workforce evolves, with projections estimating that 38% of urologists will be female by 2062 [9], it is imperative to reassess how well currently available surgical tools meet the need of our diversifying workforce.

In this study, we aimed to evaluate the relationship between surgeons’ hand size, glove fit, gender and their perceived ease of using various urologic surgical instruments. We further sought to assess the impact of these factors on instrument use -related discomfort or injury. By surveying urologists across diverse practice settings, we aimed to identify specific instruments associated with ergonomic challenges and gather recommendations for potential design modifications to improve instrument fit among urologists.

## Materials and methods

An anonymous web-based survey was developed using the Institution’s Research Electronic Data Capture database. The survey was sent to members of the Endourology Society and the Society of Women in Urology as well as various urology programs across the United States. The survey consisted of three main sections: The first section consisted of demographic information, including gender, age, ethnicity, hand dominance, practice setting, glove size and perception of glove fit. The second section explored the extent to which participants believed their hand size and hand strength impacted their ability to use surgical instruments efficiently. The ease of use of different surgical instruments as well as need for making adjustments to use them effectively were also explored in this section. Respondents were asked to report the number of cases performed monthly in each category (open, endourology, laparoscopic, robotic). If more than one case was performed per month, they were given the option to rate the instrument fit. The final section examined the level of discomfort experienced when using various surgical instruments, any interventions taken due to discomfort/injury and respondents’ recommendations for potential redesign of surgical instruments. For each instrument where respondents reported difficulty, various options and free text fields were provided. Participants completed the survey between March 2024 and May 2024.

### Statistical analysis

All statistical analyses were conducted using R (version 4.4.1). Participants missing responses to the question on glove fit or missing glove size (used as a proxy for hand size) were excluded from the analysis. Glove sizes were categorized as ≤ 6.5 versus > 6.5 to enable comparisons with prior studies defining small hands and large hands by glove size since this classification is commonly used in the literature [2, 3].

Descriptive statistics were used to summarize surgeon demographics, with continuous variables reported as mean and standard deviation (SD), and categorical variables summarized as frequencies and percentages. Comparative analyses were conducted to evaluate associations between surgeon characteristics (e.g., gender, glove size) and outcome variables. Chi-squared tests were used to compare categorical variables, with Fisher’s exact test applied when expected cell counts were below 5. Independent sample t-test was used to compare continuous variables (e.g., age, height, weight) when normality assumptions were met, otherwise the Wilcoxon rank-sum was applied. All results used a significance level/ type 1 error of 0.05.

## Results

### Demographics

The survey received 277 responses. For the purpose of gender-based analysis, two respondents who selected “Non-binary” and “Prefer not to say” were excluded from the study due to sample size of one which limited the ability to conduct meaningful analysis, resulting in a final analytic sample of 275 participants. 152/275 (55%) were female whereas 123/275 (44%) were male. The mean age of participants was 43 years (± 11.37). Females were significantly younger than male (39.8 ± 9.4 versus 46.9 ± 12.3, *P* < 0.001). Most of our participants 152/208 (73%) were practicing urologists whereas 56/208 (27%) were still in training (Table 1). Most participants were right-handed (91.5%). Hand dominance was not significantly associated with glove fit (*p* = 0.8). Among right-handed respondents, 77% reported using their dominant (right) hand to hold instruments during procedures whereas only 55% of left-handed participants used their left hand to hold the instruments.

### Glove fit

**Fig. 1:**
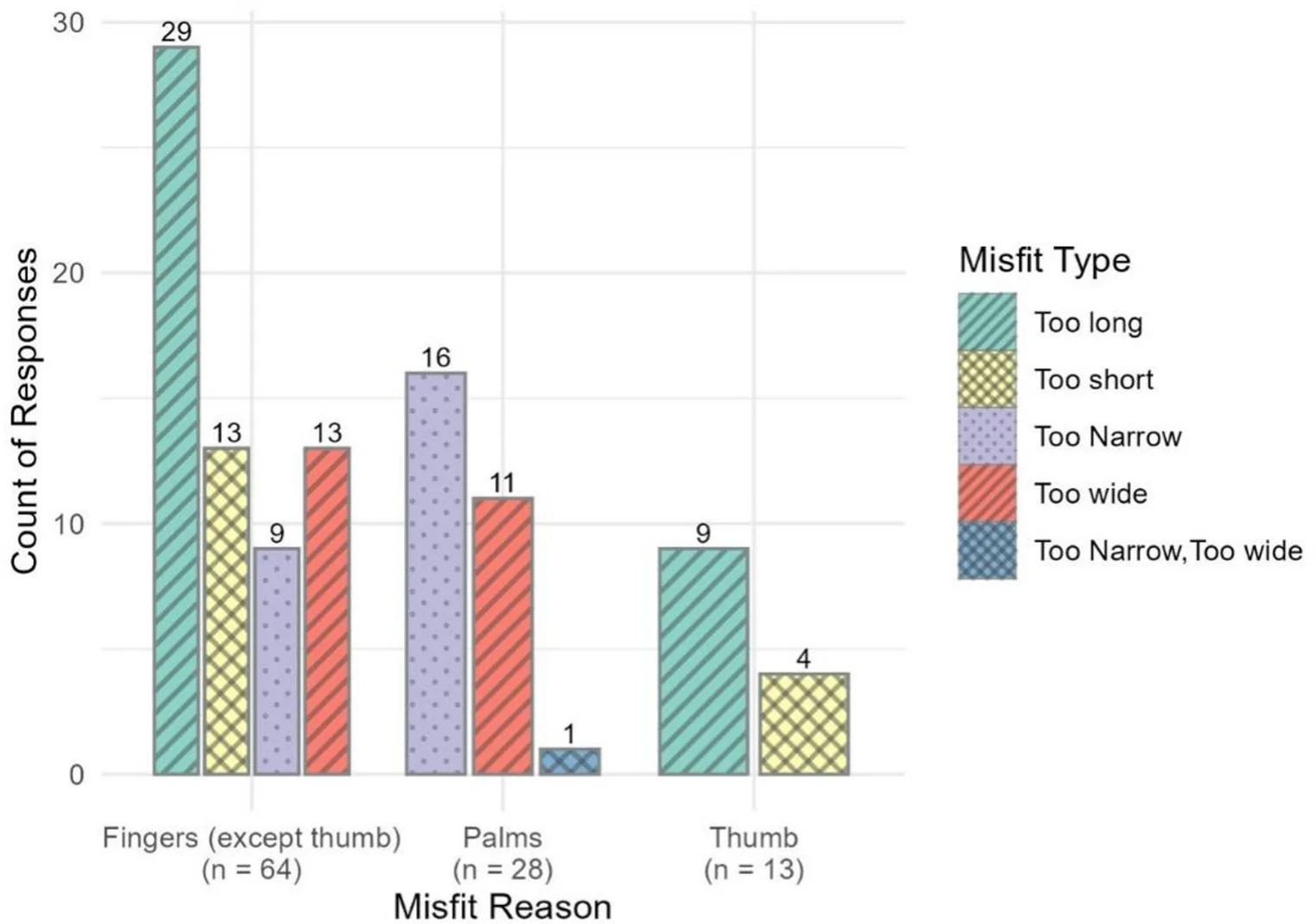
Distribution of reported reasons for glove misfit among respondents.

One quarter of participants reported glove fit issues (72/275, 26%), which was significantly higher among female compared to male respondents (32% versus 16%, *p* < 0.001) and among urologists with smaller glove size (glove size ≤ 6.5) compared to larger glove size (glove size > 6.5) (69.4% versus 27.7%, *p* < 0.001). Among urologists who reported glove fit issues, the majority (91.4%) indicated that the problem involved fingers other than the thumb. whereas 40% indicated palms as the glove fit issue and 18.6% reported thumb as the glove fit issue (Fig. 1).

### Ease of instrument use

When asked about the influence of hand size and strength on their ability to use surgical instruments, 49% of participants reported that hand size affected their ability to a great or some extent, while 62.6% indicated that hand strength had a similar impact. In terms of instrument adjustment during surgery, 52.7% reported needing to adjust their surgical instruments while operating (25.4% need adjustments at least once a week, 20.9% do so at least once every operative day and 6.3% make adjustments in every surgery).

**Fig. 2:**
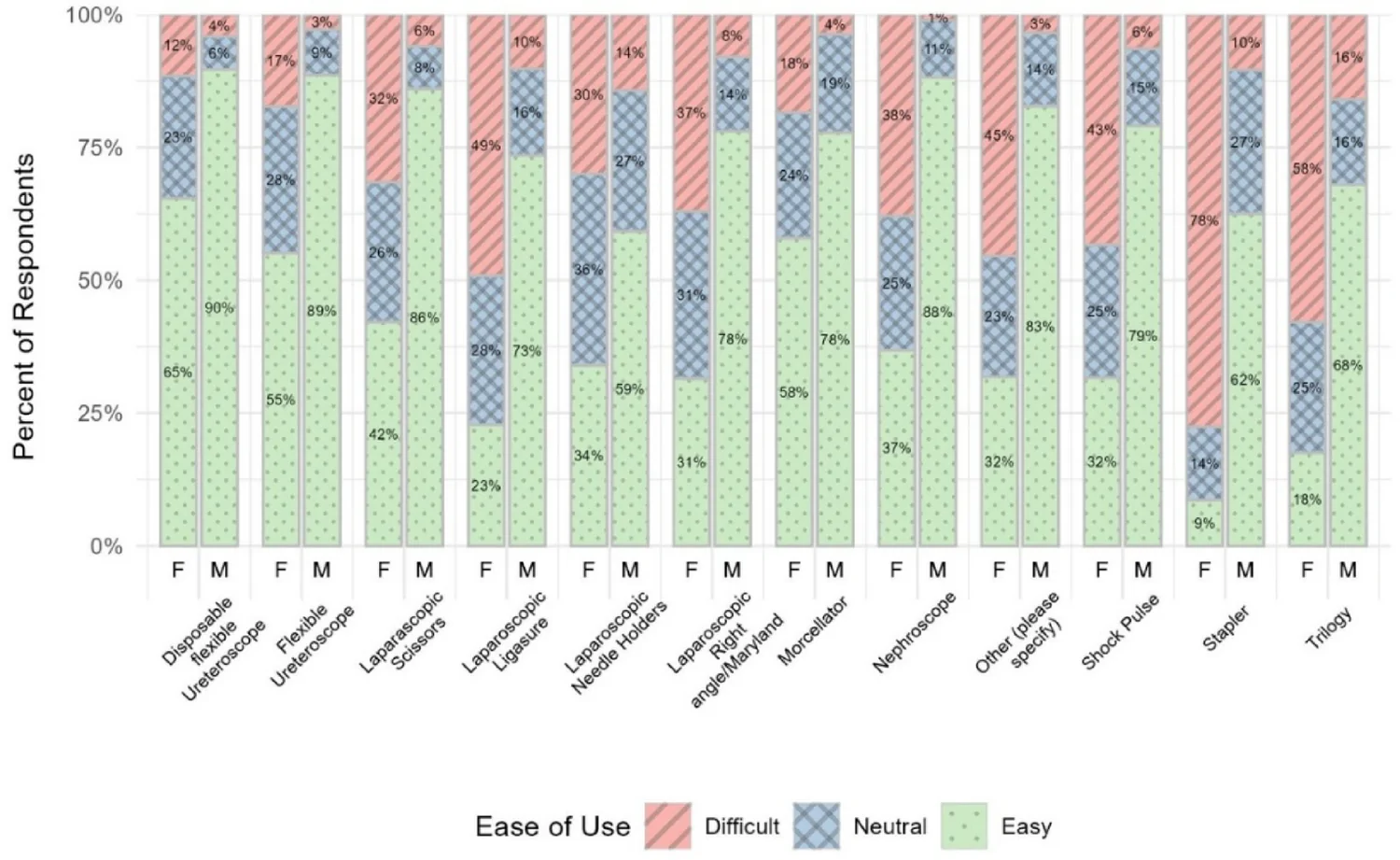
Ease-of-use ratings by gender and instrument.

**Fig. 3:**
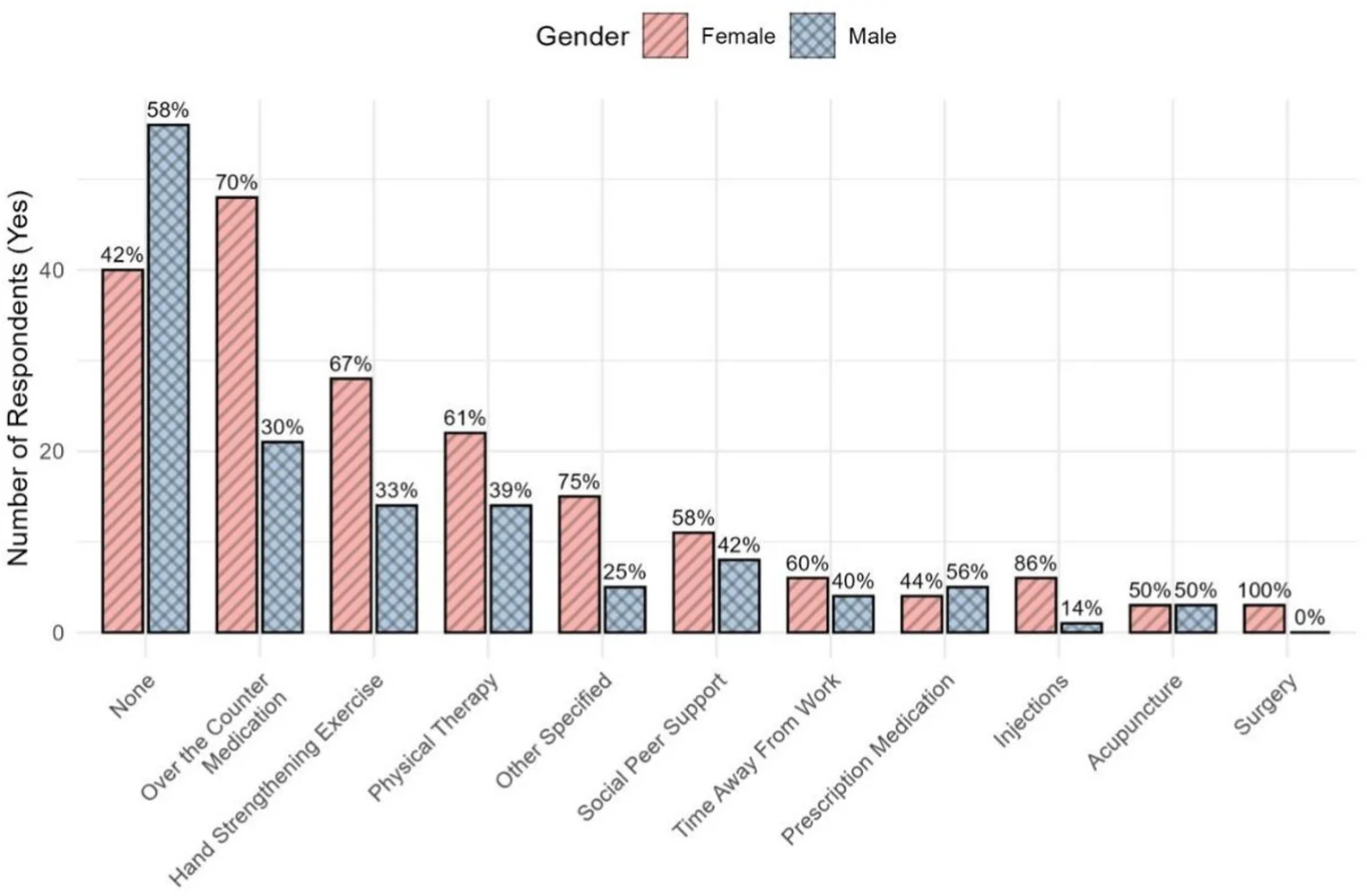
Interventions used to manage discomfort, by gender.

Out of all instruments listed in Fig. 2, surgical staplers and the Trilogy™ lithotriptor were the hardest instruments for urologists to use. Female urologists found all surgical instruments harder to use compared to their male counterparts (Fig. 2). Out of 275 participants, 113 (41%) reported using some form of intervention due to discomfort and/or injury related to the use of a surgical instrument. Female respondents were more likely than male respondents to use an intervention (71/152, 47% vs. 42/123, 34%; *p* = 0.047). 25% of participants reported having to use over the counter medication (OTC) due to discomfort/injury using a surgical instrument, which was significantly higher among females compared to males (32% versus 17%, *p* = 0.008) (Fig. 3). Most participants advocated for redesign of stapler (76%) followed by Ligasure (66%), laparoscopic needle holder (59%), Trilogy (47.7%) and flexible reusable ureteroscope (45%). Endorsing instrument redesign was significantly higher among female urologists and urologists with small hands (Glove size = < 6.5) compared to males and those with large hands (Glove size > 6.5) (Table 2). Participants were asked to rate their discomfort level when using various surgical instruments. Female urologists self-reported significantly more discomfort using the flexible ureteroscope (*p* < 0.001), disposable flexible ureteroscope (*p* = 0.003), nephroscope (*p* < 0.001), stapler (*p* < 0.001) Ligasure (*p* = 0.001), laparoscopic needle holders (*p* = 0.011), laparoscopic right angle (*p* = 0.005), Trilogy **(***p* < 0.001) and ShockPulse™ (*p* < 0.001). The highest discomfort reported among all participants regardless of gender was reported with using Trilogy (28.2%) followed by flexible reusable ureteroscope (26.8%) (Table 3). When stratified by glove size as a proxy for hand size, significant differences in level of discomfort were found between large glove size users and small glove size users when using the following instruments: flexible reusable ureteroscope (*p* < 0.001), disposable flexible ureteroscope (*p* = 0.003), nephroscope (*p* < 0.001), stapler (*p* < 0.001), laparoscopic Ligasure (*p* < 0.001), laparoscopic scissors (*p* = 0.031), laparoscopic right angle/Maryland (*p* = 0.007), Trilogy (*p* < 0.001) and ShockPulse (*p* = 0.01). Discomfort when using a morcellator (*p* = 0.6) and laparoscopic needle holder (*p* = 0.057) did not differ significantly among urologists with smaller and larger glove size. When asked about the importance of surgical instrument design, 97.73% reported this as an important issue that instruments be designed or re-designed to accommodate surgeons of all hand sizes and strengths.

## Discussion

Our findings indicate that female and small-handed urologists found all surgical instruments harder to use compared to their male and large-handed counterparts. This aligns with findings from Weinreich et al. who evaluated a large convenience sample of proceduralists and similarly reported that having a glove size ≤ 6.5 and being female increased the odds of reporting trouble using instruments [2]. Adams et al. similarly reported that female surgical trainees had the most difficulty handling the stapler and Ligasure among the laparoscopic instrument [10]. The stapler, specifically has been reported to be one of the most difficult instruments to use among laparoscopic surgeons in general [3]. In our study, we also found staplers and Trilogy™ to be the most difficult instruments to use by urologists. The majority of our respondents advocated for redesign of instruments to improve ergonomics, particularly for the stapler, Ligasure and laparoscopic needle holder, - with female and small-handed urologists expressing a greater desire when compared to their male and large-handed counterparts. Ergonomic considerations in surgical instrument design include lighter weight and optimized weight distribution, adaptable grip sizes, reduced grip and pinch forces, more neutral wrist postures, improved handle geometry and direct surgeon engagement in the redesign process [11–13].

We interestingly found that 45% of left-handed urologists use their non dominant hand to use instruments. Being forced to use the non-dominant hand can increase muscle strain, awkward posture, and the risk of cumulative injury [14]. This further reinforces inclusive instrument design to accommodate diversity in hand dominance rather than a one-size-fits-all model.

A quarter of participants reported glove fit issues with significantly higher rates among female urologists. Ill-fitting gloves can impair manual dexterity. Tight gloves restrict movement and blood flow, leading to fatigue and impaired sensation in fingertips while loose gloves reduce grip stability and tactile feedback, making it harder to manipulate surgical instruments precisely [6, 15]. Therefore, this can further contribute to difficulty using surgical instruments.

In our cohort, female urologists and small handed urologists self-reported significantly higher discomfort while using most surgical instruments listed in Fig. 2. Surgeon discomfort not only affects the well-being of the surgeon but also can affect quality of surgical care and ultimately patient outcomes. Fram et al. found female orthopedic surgeons to have a higher rate of self-reported physical symptoms attributed to surgical instrument use compared to male surgeons. Further, among female surgeons, shorter statured surgeons and surgeons with smaller glove sizes tended to have more negative attitudes toward orthopedics surgical instrument fit [16].

We previously reported a significantly larger increase in fatigue and discomfort among female urologists compared to male urologist after performing flexible ureteroscopy. Increased glove size was found to be protective against discomfort in most body regions following flexible ureteroscopy [17]. In a recent study on ergonomic set up for prostate enucleation, female urologists reported a five-fold increase in back pain compared to male urologists [18]. These findings suggest a consistent trend toward greater discomfort and fatigue among female surgeons and those with smaller hands when using current surgical instruments.

Prevalence of musculoskeletal complaints among urologists is very high, particularly among minimally invasive urologists [19]. A global survey on urologists performing retrograde intrarenal surgery, reported that 81% experienced pain in at least one body part in the last 12 months, and 51% reported that pain limited activities of daily living [20].

Burnout is also a growing concern in the field; in 2021, 36.7% of urologists reported burnout, a slight increase from 36.2% in 2016. Notably, over the same time period, while burnout rates among male urologists declined slightly (from 36.3% to 35.2%), there was a substantial rise among female urologists (from 35.3% to 49.2%) [21]. In a study on burnout and physical discomfort among vascular surgeons, Davila et al. reported work-related physical discomfort to be correlated with burnout and lack of professional satisfaction [22]. We suggest that while burnout is likely multifactorial, poor instrument fit and subsequent physical strain might be an underappreciated contributing factor.

This study has several limitations. We were not able to calculate the response rate, as it was impossible to determine the number of individuals who received the survey invitation in this convenience sample. Participants who experienced issues with glove or instrument fit might have been more likely to participate which increases the risk of selection bias. This study is subject to recall bias, which is a potential limitation since the study was based on self-reported experience, so participants who sustained an injury which they associated with difficulty using an instrument might have inaccurately recalled or exaggerated their experience. We intentionally recruited trainees to ensure that our study cohort was inclusive and representative of the broader urologic workforce. However, in our cohort, 27% of participants were still in training, so the learning curve associated with the appropriate use of surgical instruments might affect the reported discomfort. In this study, glove size is used as an indicator of hand size even though it is known to be an imperfect surrogate for actual hand size [23]; as it was only measurable indicator of hand size that could be obtained through an online anonymous study. Although hand size is a primary contributing factor, other anthropometric indices such as grip strength could potentially affect instrument usability. Future studies are needed to investigate these variables and their potential effect on surgical ergonomics. One of the strengths of this study is a relatively high number of female participants. Female surgeons are often underrepresented in in surgical ergonomics research due to their lower overall numbers in the surgical workforce. We made an effort to recruit female urologists and trainees by collaborating with the Society of Women in Urology and by reaching out to various urology residency programs across the country with a high number of female trainees.

**Table 1:** Survey respondents demographics

Characteristic	
Age (years, mean ± SD)	43.02 ± 11.37
Height (inches mean ± SD)	67.00 ± 4.01
Weight (lbs, mean ± SD)	157.53 ± 34.99
*Gender N (%)*	
Male	152/277 (54.87)
Female	123/277 (44.40)
Nonbinary	1/277 (0.36)
Prefer not to answer	1/277 (0.36)
*Race N (%)*	
White/Caucasian	130/201 (64.68%)
Asian/Pacific Islander	33/201 (16.42%)
Black or African American	14/201 (6.97%)
Prefer not to disclose	14/201 (6.97%)
Two or more races	4/201 (1.99%)
Other	6/201 (2.99%)
*Ethnicity N (%)*	
Hispanic	11/198 (5.56%)
Non-Hispanic	168/198 (84.85%)
Prefer not to disclose	19/198 (9.60%)
*Glove size N (%)*	
>6.5	143/272 (52.57)
≤6.5	129/272 (47.43)
*Average open procedures/month N (%)*	
16 or more	31/260 (11.92%)
11–15	19/260 (7.31%)
6–10	70/260 (26.92%)
1–5	107/260 (41.15%)
None	33/260 (12.69%)
*Average laparoscopy procedures/month N (%)*	
11–15	3/260 (1.15%)
6–10	14/260 (5.28%)
1–5	100/260 (38.46%)
None	143/260 (55.00%)
*Average endourology procedures/month N (%)*	
16 or more	114/260 (43.85%)
11–15	55/260 (21.15%)
6–10	39/260 (15.00%)
1–5	44/260 (16.92%)
None	8/260 (3.08%)
*Average robotic procedures/month N (%)*	
16 or more	10/260 (3.85%)
11–15	14/260 (5.38%)
6–10	39/260 (15.00%)
1–5	88/260 (33.85%)
None	109/260 (41.92%)
*Years in practice N (%)*	
20 or more years	44/208 (21.15%)
11–19 years	43/208 (20.67%)
6–10 years	33/208 (15.87%)
1–5 years	43/208 (20.67%)
Less than one year	11/208 (5.29%)
Not applicable (still in training)	43/208 (20.67%)
*Current position N (%)*	
Practicing urologist	152/208 (73.08%)
Urology resident	35/208 (16.83%)
Fellow in urologic subspecialty	19/208 (9.13%)
Research fellow	1/208 (0.48%)
*Institution region N (%)*	
North America	165/207 (79.7%)
Europe	18/207 (8.7%)
Asia	12/207 (5.8%)
South America	7/207 (3.4%)
Africa	3/207 (1.5%)
Australia and Oceania	2/207 (0.97%)

Statistical significance was defined as *p* < 0.05.

**Table 2:** Participant desire for instrument redesign

Instrument	Responses by gender and hand size	(%)	*P*-value
Reusable flexible ureteroscope	*Gender*		
	Female	53.5	**0.02**
	Male	36.2	
	*Hand size*		
	Small	53.3	0.08
	Large	40.2	
Shock pulse	*Gender*		
	Female	41.7	**0.01**
	Male	12.9	
	*Hand size*		
	Small	23.9	0.06
	Large	10.9	
Disposable flexible ureteroscope	*Gender*		
	Female	32.7	0.54
	Male	23.7	
	*Hand size*		
	Small	31.3	0.88
	Large	30.4	
Nephroscope	*Gender*		
	Female	40.0	0.05
	Male	20.4	
	*Hand size*		
	Small	94.4	**0.03**
	Large	42.6	
Trilogy	*Gender*		
	Female	54.4	0.31
	Male	42.7	
	*Hand size*		
	Small	60.0	1.0
	Large	61.1	
Stapler	*Gender*		
	Female	110.3	**< 0.0001**
	Male	35.4	
	*Hand size*		
	Small	112.0	**< 0.0001**
	Large	46.3	
Laparoscopic needle holders	*Gender*		
	Female	74.0	0.23
	Male	44.9	
	*Hand size*		
	Small	65.3	0.18
	Large	46.4	
Laparoscopic ligasure	*Gender*		
	Female	101.8	**< 0.0001**
	Male	24.5	
	*Hand size*		
	Small	116.7	**< 0.0001**
	Large	38.2	
Morcellator	*Gender*		
	Female	34.2	0.64
	Male	14.8	
	*Hand size*		
	Small	27.2	0.10
	Large	12.7	
Laparoscopic scissors	*Gender*		
	Female	59.6	**0.00**
	Male	24.0	
	*Hand size*		
	Small	65.3	**0.00**
	Large	17.3	
Laparoscopic right angle/maryland	*Gender*		
	Female	57.4	**0.00**
	Male	20.0	
	*Hand size*		
	Small	52.8	**0.00**
	Large	19.4	

**Table 3:** Instrument use discomfort

Instrument	Overall discomfort (%)	Female discomfort (%)	Male discomfort (%)	*P*-value
*Reusable flexible ureteroscope*				
Always/often	26.8	37.6	16.7	**< 0.001**
Sometimes	36.2	39.3	32.3	
Rarely/never	37.1	23.1	54.2	
*Shock pulse*				
Always/often	21.8	35.8	8.77	**< 0.001**
Sometimes	23.6	28.3	19.3	
Rarely/never	54.5	35.8	71.9	
*Disposable flexible ureteroscope*				
Always/often	17.1	24.5	9.20	**0.003**
Sometimes	33.7	37.2	29.9	
Rarely/never	49.2	38.1	60.9	
*Nephroscope*				
Always/often	17.5	32.1	2.43	**< 0.001**
Sometimes	28.3	29.8	26.8	
Rarely/never	54.2	38.1	70.7	
*Trilogy*				
Always/often	28.2	52.1	11.6	**< 0.001**
Sometimes	25.6	18.8	30.4	
Rarely/never	46.2	29.2	58.0	
*Stapler*				
Always/often	19.8	34.7	2.38	**< 0.001**
Sometimes	18.7	24.5	11.9	
Rarely/never	61.5	40.8	85.7	
*Laparoscopic needle holders*				
Always/often	15.3	23.3	7.14	**0.011**
Sometimes	29.4	39.5	19.0	
Rarely/never	55.3	37.2	73.8	
*Laparoscopic ligasure*				
Always/often	10.1	17.0	2.38	**0.001**
Sometimes	30.3	42.6	16.7	
Rarely/never	59.6	40.4	81.0	
*Morcellator*				
Always/often	8.43	13.5	4.35	0.5
Sometimes	19.3	13.5	23.9	
Rarely/never	72.3	73.0	71.7	
*Laparoscopic scissors*				
Always/often	14.0	22.4	4.55	0.13
Sometimes	23.7	24.5	22.7	
Rarely/never	62.4	53.1	72.7	
*Laparoscopic right angle/ maryland*				
Always/often	14.3	21.3	6.82	**0.005**
Sometimes	25.3	36.2	13.6	
Rarely/never	60.4	42.6	79.5	

Statistical significance was defined as *p* < 0.05.

Urology has traditionally been a male-dominant field. Therefore, gender disparity in surgical ergonomics, particularly instrument fit, has been poorly explored in urology. However, our workforce is changing as the representation of female urologists are increasing [24]. Workforce modeling projects that the total number of female urologists will grow 2.18-fold from 2020 to 2060 with an absolute increase of 1,615 urologists. With the pending urology workforce shortage, preserving the safety and career longevity of our workforce is more critical than ever. Current estimates suggest the total cost of replacing a physician lost to injury or burnout is $250k–$1 M [USD], sometimes two to three times the annual salary of the physician who left [25]. Historically, surgical instruments were designed by men for male surgeons [26] Therefore, instrument design has been slow to adapt with the changing urology workforce. However, increasing attention to ergonomic feedback has recently driven meaningful redesign. A recent example is the evolution of the Swiss LithoClast^®^ Trilogy system to the newly introduced, lighter Swiss LithoClast^®^ Trilogy Max launched in 2024 which has been shown to reduce the force applied by the surgeon during the procedure while maintaining equal efficiency [12].

## Conclusions

Our findings suggest that currently available urologic instruments do not fit a large subset of urologists. We also found that one in four urologists operates with surgical gloves that don’t fit their hands properly. Urologists reported significant discomfort while using various surgical instruments. Nearly 40% of our respondents had to use some sort of intervention to deal with the discomfort or injury caused by using surgical instruments. The majority of our sample reported having to make intraoperative adjustments in order to use the instruments effectively.

This study highlights that many urologists, predominantly female and small-handed urologists, struggle with instrument fit. Our data supports the need for potential redesign of many urologic instruments. 97% of respondents agreed that it is important for surgical instruments to be designed or re-designed to accommodate diverse hand sizes and strengths. We advocate for involvement of urologists from all demographics in every stage of surgical instrument design from concept creation to prototype testing all the way to post marketing evaluation. In an era where individualized medicine is shaping the future of patient care, size adjustable instruments or customized 3D printed gloves no longer seem far-fetched.

## Data Availability

The data is available upon request.
